# Ambient Air Pollution and Hospitalizations for Ischemic Stroke: A Time Series Analysis Using a Distributed Lag Nonlinear Model in Chongqing, China

**DOI:** 10.3389/fpubh.2021.762597

**Published:** 2022-01-18

**Authors:** Hao Chen, Zheng Cheng, Mengmeng Li, Pan Luo, Yong Duan, Jie Fan, Ying Xu, Kexue Pu, Li Zhou

**Affiliations:** ^1^Department of Epidemiology, School of Public Health and Management, Chongqing Medical University, Chongqing, China; ^2^College of Medical Informatics, Chongqing Medical University, Chongqing, China

**Keywords:** air pollution, short-term, ischemic stroke, hospitalizations, DLNM

## Abstract

Short-term exposure to air pollution has been associated with ischemic stroke (IS) hospitalizations, but the evidence of its effects on IS in low- and middle-income countries is limited and inconsistent. We aimed to quantitatively estimate the association between air pollution and hospitalizations for IS in Chongqing, China. This time series study included 2,299 inpatients with IS from three hospitals in Chongqing from January 2015 to December 2016. Generalized linear regression models combined with a distributed lag nonlinear model (DLNM) were used to investigate the impact of air pollution on IS hospitalizations. Stratification analysis was further implemented by sex, age, and season. The maximum lag-specific and cumulative percentage changes of IS were 1.2% (95% CI: 0.4–2.1%, lag 3 day) and 3.6% (95% CI: 0.5–6.7%, lag 05 day) for each 10 μg/m^3^ increase in PM_2.5_; 1.0% (95% CI: 0.3–1.7%, lag 3 day) and 2.9% (95% CI: 0.6–5.2%, lag 05 day) for each 10 μg/m^3^ increase in PM_10_; 4.8% (95% CI: 0.1–9.7%, lag 4 day) for each 10 μg/m^3^ increase in SO_2_; 2.5% (95% CI: 0.3–4.7%, lag 3 day) and 8.2% (95% CI: 0.9–16.0%, lag 05 day) for each 10 μg/m3 increase in NO_2_; 0.7% (95% CI: 0.0–1.5%, lag 6 day) for each 10 μg/m^3^ increase in O_3_. No effect modifications were detected for sex, age, and season. Our findings suggest that short-term exposure to PM_2.5_, PM_10_, SO_2_, NO_2_, and O_3_ contributes to more IS hospitalizations, which warrant the government to take effective actions in addressing air pollution issues.

## Introduction

Stroke is a predominant public health concern in the world and the leading cause of death in China, which caused 6.2 million deaths and 132 million disability-adjusted life years globally in 2017 (1, 2). In China, ischemic stroke (IS) is the main subtype of stroke, accounting for 80% of all stroke events (3). Moreover, the incidence of IS has increased sustainedly in China in recent years (4), which has generated a huge burden on healthcare costs and the economy. Therefore, identification of modifiable risk factors for IS has substantial public health implications.

Many factors have been confirmed to be related to IS, including smoking, lacking of physical exercise, hypertension, obesity, and atrial fibrillation (5). In addition, increasing epidemiological evidence has shown a striking relationship between air pollution exposure and IS (6, 7). A study conducted in nine US counties demonstrated the short-term effects of PM_10_, CO, SO_2_, and NO_2_ on hospitalizations for IS (8). Another study by Tian, Y. et al. observed a significant increase in hospitalizations for IS with transient increases of PM_2.5_, SO_2_, NO_2_, O_3_, and CO in China (9). However, most of these studies were conducted in high-income countries, and the scientific evidence that generated in low- or middle-income countries was scarce, especially in China (10). Recently, data from the National Epidemiological Survey of Stroke in China showed that the estimated mortality-to-incidence ratio (MIR) of stroke is the highest in the southwest and the lowest along the eastern and southern coasts; the proportion of registered medical doctors per 1,000 of the population is the highest in northern and eastern China and the lowest in the southwest (4). Furthermore, the geographical regions with high correlation between IS mortality and PM_2.5_ gradually moved from western and northern China to the southwest during 1990–2015. Therefore, it was of great importance to explore the associations between air pollution and IS in the southwest.

Chongqing is a major heavy industrialized city located in Southwest China. Heavy industry and valley basin structure contributed to some of the worst air pollution in comparison with China's other cities. Thus, it is more meaningful to study the associations between the ambient air pollution and the occurrence of IS in Chongqing.

In this study, we conducted a time series analysis to investigate the associations between short-term exposure to air pollution and daily IS hospitalizations in Chongqing, China.

## Materials and Methods

### Study Area and Health Data Collection

Chongqing is a major heavy industrialized city and one of the four municipalities in China. It is an urban city with an area of 5472.68 km^2^ and a population of greater than 8.6 million in 2017 (11).

Data on daily hospital admissions for IS in this study were retrieved from 3 tertiary-level comprehensive hospitals (The Second Affiliated Hospital of Chongqing Medical University, University-Town Hospital of Chongqing Medical University, and The Southeast Hospital) with approximately 3,500, 1,500, and 1,200 inpatient beds, respectively. The medical information was recorded on Platform of Medical Data Science Academy of Chongqing Medical University, and it included the patients' age, sex, diagnosis, dates of admission, and discharge. We identified admissions for IS (International Classification of Diseases (ICD)-10 code I63) from January 1, 2015 to December 31, 2016 according to the 10th revision of ICD-10 codes.

### Environmental Data

**Figure 1:**
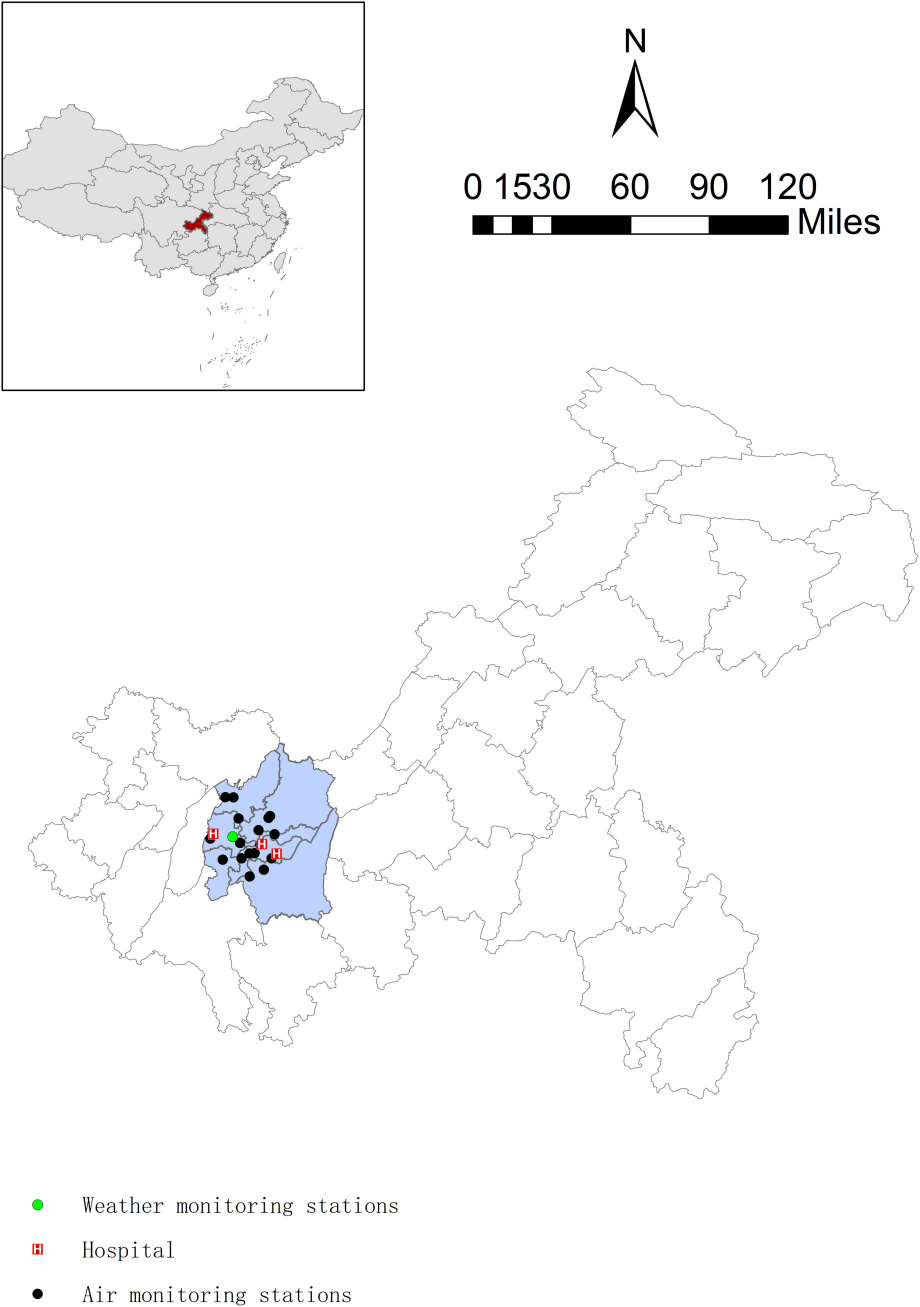
Spatial distributions of air quality monitoring stations, weather monitoring stations, and addresses of hospitals in Chongqing, China.

Data on air pollution, including levels of PM_2.5_, PM <10 μm in aerodynamic diameter (PM_10_), sulfur dioxide (SO_2_), nitrogen dioxide (NO_2_), carbon monoxide (CO), and ozone (O_3_) between January 1, 2015 and December 31, 2016, were obtained from the China Air Quality Online Monitoring and Analysis Platform. There were 17 fixed monitoring stations that measure concentrations of air pollution in Chongqing. In the Chinese air quality online monitoring system, PM_10_ and PM_2.5_ were monitored by continuous automatic β-ray monitoring method, SO_2_ and O_3_ by ultraviolet fluorescence, and NO_2_ by chemiluminescence. These measurements were completed in adherence to the China National Quality Control (GB3095-2012) protocol. We derived 24-h mean concentrations of these pollutants in Chongqing to represent each individual's daily exposure levels except for ozone (by averaging 8-h maximum values). Weather conditions including daily mean temperature and mean relative humidity were sourced from the National Meteorological Information Center of China. All the monitoring stations and hospitals were located in urban areas (Figure 1).

### Statistical Analysis

Spearman's correlation coefficient was applied to explore the correlation between air pollutants and meteorological factors. For the time series analysis, we used generalized linear model combined with a distributed lag non-linear model (DLNM) to investigate both non-linear and delayed effects of air pollutants on daily hospitalizations for IS. As daily IS hospital admissions data being quantitative data, belonging to small probability events, and conforming to Poisson distribution, there a Poisson regression models allowing for overdispersion was used. The model is as follows:


$$\begin{array}{l} Log\left(Yt\right)=\alpha +cb\left(Xt,\;lag\right)+cb\left(Mean\;Temt,lag\right)\\ \;+ns\left(RHt,df=3\right)+ns\left(Timet,df=7*years\right)\\ \;+factor\left(DOW\right)+factor\left(holiday\right) \end{array}$$

where *t* is day of observation, *Yt* refers to the number of IS hospital admissions, *cb*(*Xt, lag*) and *cb*(*Mean Temt, lag*) indicate the matrix of air pollution and mean temperature by applying the DLNM, *Xt* and *Mean Temt* represent pollutant concentrations and mean temperature at day t, *ns*(*RHt, df* = 3) are natural cubic spline with 3 degrees of freedom for daily relative humidity, *ns*(*Timet, df* = 7*year) is 7 degrees of freedom per year, to control long-term trend and seasonality (12, 13), DOW is the day of the week, and holiday is the variable for the holiday effect. We adopt a linear and a natural cubic spline (*df* = 3) function to fit the exposure–response relationship and the lag–response relationship, respectively (13). Also, the parameter of lag–response relationship between mean temperature and IS was equal to air pollutants. Furthermore, the non-linear relationship between air pollution and IS was explored by a natural cubic spline function (*df* = 3) when the cumulative effect appears to be strongest.

Previous studies had shown that the lagged effect of air pollutants was usually short. In this study, single-day lags (from lag 0 day to lag 7 day) combined with cumulative lags (from lag 01 day to lag 07 day) were applied to evaluate the lagged effects of air pollutants. We initially performed single-pollutant model to assess the association between air pollution and IS hospitalizations, and then, the significant air pollutants were included in subsequent analysis.

We conducted stratification analysis to explore the potential effect modification by age (<75, ≥ 75 years), sex (male, female), and season [cold season, warm season). The risk estimates were expressed in terms of the percentage changes (excess risk, ER (%)] in IS hospitalizations per 10 μg/m^3^ increment of air pollutants (except that CO was per 1 mg/m^3^) and their respective 95% confidence intervals (CIs). We further evaluated the statistical significance of the differences as $\left(Qˆ1-Qˆ2\right)/\sqrt{}{SEˆ1}^{2}\;+{SEˆ2}^{2}$, such that the *Qˆ*1 and $\hat{Q}2$ represent the estimates for the two subgroups, and SE1 and SE2 represent their respective standard errors.

Sensitivity analysis was also performed to identify the robustness of the results by (a) fitting two-pollutant models. Correlation coefficient *r* > 0.60 between air pollutants was not included in multipollutant model to avoid collinearity; (b) changing the degrees of freedom in the natural cubic spline function of time (6–8 *df*) and meteorological variables (4–6 *df*). All analyses were conducted through “dlnm” and “splines” packages in R software (version 3.6.3). *p-*Value less than 0.05 was considered as statistically significant.

## Results

Basic characteristics of patients with IS, meteorological factors, and air pollution are displayed in Table 1. There was a total of 14,969 hospital admissions for cardiovascular and cerebrovascular diseases from 3 hospitals during January 1, 2015 to December 31, 2016. Overall, 2,299 patients with IS formed the basis of this study, with a daily average of 3 cases. 57.1% were men and 65.9% were patients aged ≥75 years. The mean 24-h PM_2.5_, PM_10_, SO_2_, CO, NO_2_, and 8-h maximum O_3_ concentrations were 54.41 μg/m^3^, 81.75 μg/m^3^, 14.55 μg/m^3^, 1.04 mg/m^3^, 45.05 μg/m^3^, and 68.11 μg/m^3^, respectively.

**Table 1:** Distribution of daily IS admissions, air pollutants, and meteorological factors in Chongqing, China (January 2015–December 2016).

**Variables**	**Mean±SD**	**Min**	**P10**	**P25**	**P50**	**P75**	**P90**	**P95**	**P99**	**Max**
Cardiovascular and cerebrovascular disease	20.48 ± 9.49	2.0	9.0	13.0	19.0	26.0	34.0	38.0	47.0	57.0
IS	3.15 ± 2.00	0.0	1.0	2.0	3.0	4.0	6.0	7.0	9.0	13.0
**Gender**										
Male	1.35 ± 1.23	0.0	0.0	0.0	1.0	2.0	3.0	4.0	5.0	7.0
Female	1.80 ± 1.45	0.0	0.0	1.0	2.0	3.0	4.0	5.0	6.0	7.0
**Age**										
<75	1.07 ± 1.08	0.0	0.0	0.0	1.0	2.0	3.0	3.0	4.0	6.0
≥75	2.07 ± 1.55	0.0	0.0	1.0	2.0	3.0	4.0	5.0	6.0	10.0
**Season**										
Warm	3.06 ± 1.95	0.0	1.0	2.0	3.0	4.0	6.0	7.0	8.0	13.0
Cold	3.23 ± 2.04	0.0	1.0	2.0	3.0	5.0	6.0	7.0	9.0	10.0
PM_2.5_ (μg/m^3^)	54.41 ± 30.72	10.0	25.0	34.0	46.0	66.0	93.0	114.0	164.1	212.0
PM_10_ (μg/m^3^)	81.75 ± 40.68	13.0	41.0	54.0	74.0	98.0	131.0	164.5	228.8	293.0
SO_2_ (μg/m^3^)	14.55 ± 6.59	4.0	7.0	10.0	13.0	18.0	23.0	27.0	36.4	42.0
CO (mg/m^3^)	1.04 ± 0.28	0.4	0.7	0.9	1.0	1.2	1.4	1.5	1.8	3.4
NO_2_ (μg/m^3^)	45.05 ± 12.69	16.0	30.0	36.0	44.0	53.0	62.0	69.0	79.0	96.0
O_3_ (μg/m^3^)	68.11 ± 45.38	4.0	15.0	29.0	60.0	101.0	135.0	150.5	184.3	223.0
Temperature (°C)	19.60 ± 7.47	1.2	9.6	13.0	20.1	25.2	29.4	31.4	34.3	36.2
Relative humidity (%)	75.36 ± 10.93	43.0	60.3	68.0	76.0	84.0	90.0	92.0	94.6	96.3

*PM_2.5_, particles with aerodynamic diameter <2.5 μm; PM_10_, particles with aerodynamic diameter <10 μm; SO_2_, sulfur dioxide; NO_2_, nitrogen dioxide; CO, carbon monoxide; O_3_, ozone; IS, ischemic stroke*.

The Spearman's correlation coefficients between air pollutants and meteorological factors are listed in Table 2. PM_2.5_, PM_10_, SO_2_, NO_2_, and CO were positively and moderately or strongly correlated. O_3_ exposure was negatively and weakly associated with PM_2.5_, SO_2_, and CO, whereas the correlation coefficient with PM_10_ and NO_2_ was not statistically significant. Temperature was positively associated with O_3_, and negatively associated with other air pollutants, whereas relative humidity was positively associated with CO, and negatively associated with other air pollutants.

**Table 2:** Spearman's correlation coefficients between air pollutants and meteorological factors.

**Variables**	**PM_**2.5**_**	**PM_**10**_**	**SO_**2**_**	**CO**	**NO_**2**_**	**O_**3**_**	**Temperature**	**Relative humidity**
PM_2.5_	1.00							
PM_10_	0.95**	1.00						
SO_2_	0.61**	0.68**	1.00					
CO	0.60**	0.56**	0.56**	1.00				
NO_2_	0.71**	0.74**	0.61**	0.53**	1.00			
O_3_	−0.09*	0.04	−0.15**	−0.44**	−0.04	1.00		
Temperature	−0.26**	−0.15**	−0.35**	−0.41**	−0.23**	0.73**	1.00	
Relative humidity	−0.12**	−0.29**	−0.39**	0.23**	−0.14**	−0.65**	−0.38**	1.00

PM_2.5_, particles with aerodynamic diameter <2.5 μm; PM_10_, particles with aerodynamic diameter <10 μm; SO_2_, sulfur dioxide; NO_2_, nitrogen dioxide; CO, carbon monoxide; O_3_, ozone; * p < 0.05; ** p < 0.01.

**Figure 2:**
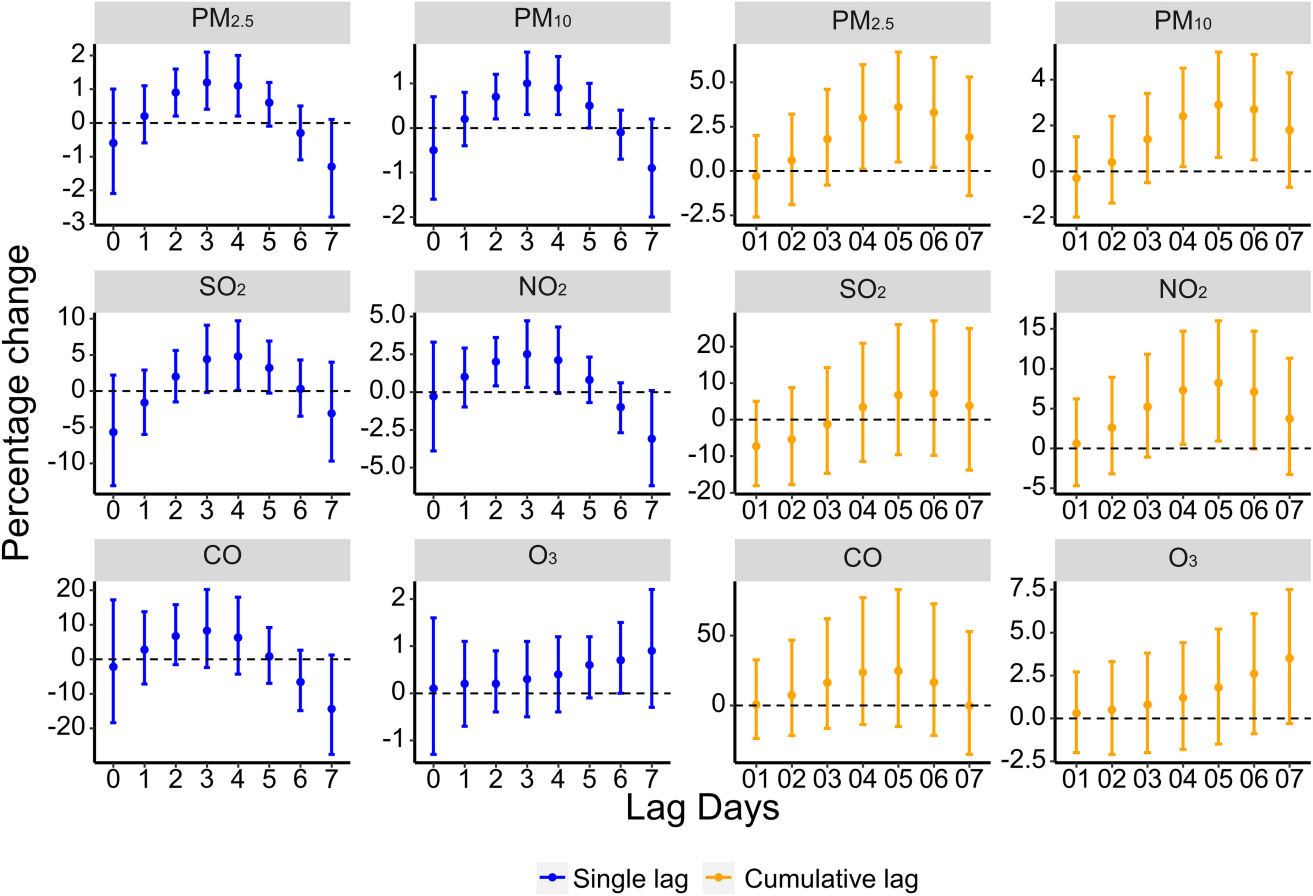
Percentage changes [excess risk, ER (%)] of IS cases for each pollutant at various lags.

Figure 2 shows that exposure to PM_2.5_, PM_10_, SO_2_, NO_2_, and O_3_ was significantly associated with IS. In single-day lag structures, exposures to PM_2.5_ (lag 2 day–lag 4 day), PM_10_ (lag 2 day–lag 5 day), SO_2_ (lag 4 day), NO_2_ (lag 2 day, lag 3 day), and O_3_ (lag 6 day) were associated with increased IS cases. The maximum lag-specific percentage changes for each 10 μg/m^3^ increase in PM_2.5_, PM_10_, SO_2_, NO_2_, and O_3_ were 1.2% (95% CI: 0.4–2.1%, lag 3 day), 1.0% (95% CI: 0.3–1.7%, lag 3 day), 4.8% (95% CI: 0.1–9.7%, lag 4 day), 2.5% (95% CI: 0.3–4,7%, lag 3 day), and 0.7% (95% CI: 0.0–1.5%, lag 6 day), respectively. In cumulative lag structures, the unfavorable effects of PM_2.5_, PM_10_, and NO_2_ occurred to lag 04 day–lag 06 day, lag 04 day–lag 06 day, and lag 04 day–lag 05 day, and the corresponding maximum percentage changes were 3.6% (95% CI: 0.5–6.7%, lag 05 day), 2.9% (95% CI: 0.6–5.2%, lag 05 day), 8.2% (95% CI: 0.9–16.0%, lag 05 day) (Supplementary Table S1), whereas no association between CO and IS had been detected.

**Figure 3:**
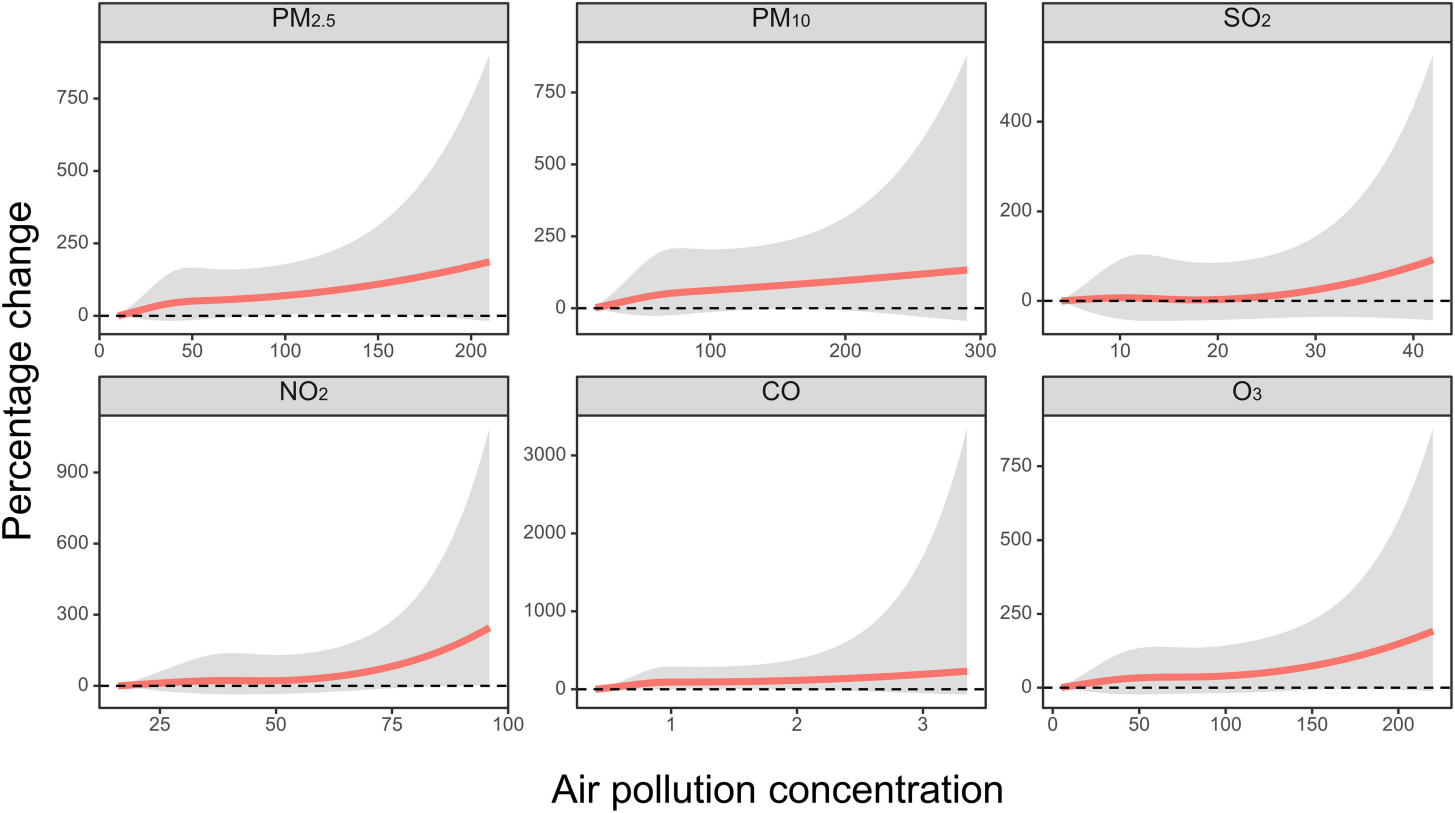
The concentration–response relationship curves between PM_2.5_ (lag 05 day), PM_10_ (lag 05 day), SO_2_ (lag 06 day), NO_2_ (lag 05 day), and O_3_ (lag 07 day) and IS hospitalizations. *X*-axis: each air pollution concentrations; *y*-axis: the percentage changes [excess risk, ER (%)] of air pollution on IS.

The exposure–response curves for PM_2.5_, PM_10_, SO_2_, NO_2_, CO, and O_3_ with hospitalizations for IS are displayed in Figure 3. The exposure–response curve for NO_2_, SO_2_, and O_3_ showed thresholds for their associations with hospitalizations for IS: 50–75 μg/m^3^ for NO_2_, 20–30 μg/m^3^ for SO_2_, and 100–150 μg/m^3^ for O_3_. The exposure–response relationship for PM_2.5_, PM_10_, and CO was almost linear.

Stratified and sensitivity analyses were performed on the basis of reaching the maximum effect of PM_2.5_, PM_10_, SO_2_, NO_2_, and O_3_ in the cumulative lag structure. The associations between air pollutant exposures and the hospitalizations for IS were evaluated in subgroups based on sex, age, and season (Table 3). For PM_2.5_, PM_10_, SO_2_, and NO_2_, the effects were still significant in cold season subgroup. In addition, both sex and the younger received adverse effects when exposed to PM_10_, whereas NO_2_ seems to be more susceptible to men and the elder. Although the percentage changes were somewhat different and some of them were insignificant, we did not find any significant effect modification across sex, age, and season (all *p* for effect modification > 0.05).

**Table 3:** Percentage changes [excess risk, ER (%)] for IS hospitalizations with per 10 μg/m^3^ increase of exposure to PM_2.5_, PM_10_, SO_2_, NO_2_, and O_3_ stratified by age, sex, and season.

**Variables**	**PM**_**2.5**_		**PM**_**10**_		**SO**_**2**_		**NO**_**2**_		**O**_**3**_	
	**ER% (95%CI)**	***p–*value**	**ER% (95%CI)**	***p–* value**	**ER% (95%CI)**	***p–* value**	**ER% (95%CI)**	***p–* value**	**ER% (95%CI)**	***p–* value**
Male	22.8 (−15.4–78.0)		3.3 (0.0–6.7)		−1.8 (−23.5–26.2)		12.6 (1.8–24.6)		−2.2 (−6.7–2.7)	
Female	9.1 (−20.6–49.9)	0.636	3.0 (0.2–5.8)	0.891	21.5 (−2.4–51.2)	0.210	7.6 (−1.5–17.4)	0.502	2.1 (−2.0–6.5)	0.184
<75	25.1 (−16.1–86.5)		4.3 (0.9–7.9)		9.8 (−15.9–43.4)		10.9 (−0.6–23.7)		−1.7 (−6.7–3.6)	
≥75	8.5 (−19.1–45.6)	0.576	2.5 (−0.1–5.1)	0.398	11.1 (−9.3–36.0)	0.947	9.0 (0.5–18.2)	0.805	1.2 (−2.6–5.1)	0.383
Warm	5.8 (−1.8–14.0)		3.4 (−1.5–8.5)		−1.7 (−28.4–35.0)		9.6 (−3.2–24.1)		1.1 (−3.7–6.2)	
Cold	3.8 (0.1–7.7)	0.651	3.5 (0.7–6.4)	0.964	21.9 (0.7–47.7)	0.255	15.2 (4.9–26.4)	0.530	3.6 (−3.3–10.9)	0.579

*PM_2.5_, particles with aerodynamic diameter <2.5 μm; PM_10_, particles with aerodynamic diameter <10 μm; SO_2_, sulfur dioxide; NO_2_, nitrogen dioxide; O_3_, ozone; CI, confidence intervals*.

Table 4 provides the results of the two-pollutant model, and further adjustment for other pollutant exposures did not materially change the associations between PM_2.5_, PM_10_, SO_2_, NO_2_, andO_3_ exposures and IS. Results of the sensitivity analysis (Supplementary Table S2) indicated that the effect estimates of the association between air pollution and IS hospitalizations were not substantially affected with the use of alternative *df* value for time (6–8 per year), temperature (3–6), and relative humidity (3–6), although some of the associations became insignificant for temperature (*df* = 6).

**Table 4:** Percentage changes [excess risk, ER (%)] of IS for each pollutant in two-pollutant model.

		**ER% (95%CI)**	***p-* value**
PM_2.5_	NULL	3.6 (0.5–6.7)	
	O_3_	2.7 (−0.5–6.1)	0.715
PM_10_	NULL	2.9 (0.6–5.2)	
	CO	2.7 (0.0–5.4)	0.907
	O_3_	2.3 (−0.2–4.8)	0.759
SO_2_	NULL	7.1 (−9.8–27.0)	
	CO	3.4 (−13.5–23.7)	0.787
	O_3_	0.6 (−16.2–20.7)	0.626
NO_2_	NULL	8.2 (0.9–16.0)	
	CO	7.0 (−1.2–15.8)	0.838
	O_3_	6.8 (−1.0–15.1)	0.789
O_3_	NULL	3.5 (−0.3–7.5)	
	PM_2.5_	2.6 (−1.4–6.9)	0.778
	PM_10_	2.2 (−2.0–6.6)	0.672
	SO_2_	3.4 (−0.6–7.6)	0.971
	NO_2_	3.0 (−1.1–7.3)	0.888
	CO	3.3 (−0.5–7.3)	0.970

*PM_2.5_, particles with aerodynamic diameter <2.5 μm; PM_10_, particles with Aerodynamic diameter <10 μm; SO_2_, sulfur dioxide; NO_2_, nitrogen dioxide; O_3_, ozone; CI, confidence intervals*.

## Discussion

There is growing interest in the associations between ambient air pollution and risk of IS, but data on the associations in low- or middle-income cities are still limited, especially in Southwest China. Chongqing is mainly associated with heavy industry with a valley basin structure, which has a great impact on ambient air pollution. Therefore, we conducted this time series study combining with DLNM to evaluate the short-term effects of ambient air pollutants on hospitalizations for IS in Chongqing. The data obtained in this analysis indicated that short-term exposure to PM_2.5_, PM_10_, NO_2_, O_3_, and SO_2_ was significantly associated with increased hospitalizations for IS. Age, sex, and season did not appear to significantly modify the associations between short-term exposure to air pollution and IS onset. The sensitivity analysis showed a robustness of the pollutant model results.

In single-pollutant models, we found that each 10 μg/m^3^ increase in PM_2.5_, PM_10_, SO_2_, NO_2_, and O_3_ was associated with a 1.2, 1.0, 4.8, 2.5, and 0.7% increase in IS hospitalizations, respectively, which were similar to several previous studies (14–16). For example, Hu's study conducted in Yancheng city has shown that IS hospitalizations increased 1.06% (95% CI: 0.21–1.91%) per 10 μg/m^3^ increase in PM_2.5_ (14). Another multicity study reported that per 10 μg/m^3^ evaluation of NO_2_ was associated with 2.6% change in hospitalizations for IS (15). Wellenius *et al*. investigated the association of air pollution with hospitalizations for IS in nine US cities, with a cohort restricted to patients aged above 65 years. They observed that an elevation of IQR in PM_10_ (22.96 μg/m^3^), SO_2_ (6.69 μg/m^3^), and NO_2_ (11.93 μg/m^3^) concentrations was associated with 1.03, 1.35, and 2.94% increases in hospitalizations for IS, respectively (8). Another study conducted in Guangzhou indicated that each IQR increase of PM_2.5_ (41 μg/m^3^), O_3_ (99 μg/m^3^), SO_2_ (15 μg/m^3^), and NO_2_ (44 μg/m^3^) corresponded to an RR value of 1.0272, 1.0173, 1.0344, 1.0423, respectively (17). In addition, a nationwide time series analysis in China indicated that each increase of 10 μg/m^3^ in PM_2.5_, SO_2_, and NO_2_ was associated with a 0.34, 1.37, and 1.82% increase in hospitalizations for IS (9). Their studies suggest that the major components of air pollutants vary significantly from region to region, which does affect the occurrence of IS in different cities.

In China, the evidence of the effect of CO exposure on IS risk is still controversial. A study conducted in Taiwan found that CO was significantly and positively associated with IS hospitalizations in the single-pollutant model, but it became insignificant in the multipollutant model (18). However, research from Hong Kong observed a negative association between ambient CO concentrations and stroke hospitalizations (19). In addition, several other studies in China found no association between CO and IS risk (15, 20), which was consistent with our results. The inconsistency of the results on the associations between CO and IS might be attributable to variations in air pollution levels, outcome definitions, weather conditions, population susceptibility, and sociodemographic characteristics across studies.

The identification of potentially susceptible subpopulations has significant implications for public health. Some studies did not identify any effect modification by sex and age between air pollution and IS (21–23), which were in line with our study. In addition, this study found no effect modification by season, whereas the adverse effects of PM_2.5_, PM_10_, NO_2_, and SO_2_ remained significant in cold season. This may be attributable to a high number of foggy days during cold season in Chongqing. During our study period, the mean AQI in Chongqing (80.75) was higher than a less foggy city, such as Guangzhou (68.79), which was similar to Chongqing in population and land area (24).

The biological mechanism of air pollution-induced IS has not been fully investigated. Several possible mechanisms have been proposed to be related to the inflammation (25), oxidative stress (26), abnormal lipid metabolism (7, 27), and autonomic dysfunction (7). For example, PM_2.5_ can trigger the release of pro-inflammatory mediators causing systemic inflammation, which impaired blood–brain barrier (BBB) stability and induces to the generation of ROS and oxidative stress (28). Inhalation of SO_2_ can affect heart rate variability, increase oxidation, and exacerbate blood clotting and thrombosis formation (29). In addition, a study showed that exposure to O_3_ and particulate pollutants increased endothelin 1, leading to vascular endothelial dysfunction and subsequent brain damage (30).

Our study has some potential limitations. First, small sample sizes might lead to lower statistical power; therefore, subsequent studies with large sample sizes would be expected. Second, city-level concentration of air pollution rather than individual exposure was utilized as the exposure concentration, which might underestimate the effect of air pollution. Third, we selected only 3 hospitals in one single city, so the generalization of the results requires caution. Finally, although we adjusted for several confounders such as seasonality, day of week, public holiday, and weather conditions, there might exist some other confounding factors.

## Conclusions

This study suggests that short-term exposure to PM_2.5_, PM_10_, NO_2_, O_3_, and SO_2_ was associated with increased hospitalizations for IS in Chongqing, China. Our study provides new evidence on the association between air pollution and IS. Further studies are warranted to help government effectively reduce the burden of air pollution.

## Data Availability Statement

The raw data supporting the conclusions of this article will be made available by the authors, without undue reservation.

## Ethics Statement

The studies involving human participants were reviewed and approved by Ethics Committee of Chongqing Medical University in Chongqing, China. Written informed consent to participate in this study was provided by the participants' legal guardian/next of kin.

## Author Contributions

HC wrote the manuscript and analyzed the data. PL and YD collected and inputted the data. JF, YX, ML, ZC, KP, and LZ reviewed the results and provided guidelines for presentation and interpretation. All authors have read and approved the final manuscript.

## Funding

This study was funded by Intelligent Medicine Research Project of Chongqing Medical University (Number: ZHYX202026) and Intelligent Medicine Research Project for Graduate Students of Chongqing Medical University (Number: YJSZHYX202018).

## Conflict of Interest

The authors declare that the research was conducted in the absence of any commercial or financial relationships that could be construed as a potential conflict of interest.

## Publisher's Note

All claims expressed in this article are solely those of the authors and do not necessarily represent those of their affiliated organizations, or those of the publisher, the editors and the reviewers. Any product that may be evaluated in this article, or claim that may be made by its manufacturer, is not guaranteed or endorsed by the publisher.
