# Supplementary material for: Ambient Air Pollution and Hospitalizations for Ischemic Stroke: A Time Series Analysis Using a Distributed Lag Nonlinear Model in Chongqing, China
Source: Front Public Health. 2022 Jan 18;9:762597. doi: 10.3389/fpubh.2021.762597 (PMC8804166; doi:10.3389/fpubh.2021.762597)
Supplement: Supplementary file 1 [file Table_1.DOCX]

Supplementary Material

Table S1. Percentage changes (excess risk, ER (%)) with 95%CI of ischemic stroke hospitalizations for each pollutant at various lag days

|  | PM_2.5_ | PM_10_ | SO_2_ | NO_2_ | CO | O_3_ |
| --- | --- | --- | --- | --- | --- | --- |
| lag0 | -0.6 (-2.1-1.0) | -0.5 (-1.6-0.7) | -5.7 (-13.1-2.2) | -0.3 (-3.9-3.3) | -2.2 (-18.4-17.2) | 0.1 (-1.3-1.6) |
| lag1 | 0.2 (-0.6-1.1) | 0.2 (-0.4-0.8) | -1.6 (-6.0-2.9) | 1.0 (-1.0-2.9) | 2.8 (-7.2-13.8) | 0.2 (-0.7-1.1) |
| lag2 | 0.9 (0.2-1.6) | 0.7 (0.2-1.2) | 2.0 (-1.5-5.6) | 2.0 (0.4-3.6) | 6.7 (-1.6-15.8) | 0.2 (-0.4-0.9) |
| lag3 | 1.2 (0.4-2.1) | 1.0 (0.3-1.7) | 4.4 (-0.2-9.1) | 2.5 (0.3-4.7) | 8.3 (-2.4-20.2) | 0.3 (-0.5-1.1) |
| lag4 | 1.1 (0.2-2.0) | 0.9 (0.3-1.6) | 4.8 (0.1-9.7) | 2.1 (-0.1-4.3) | 6.3 (-4.3-18.0) | 0.4 (-0.4-1.2) |
| lag5 | 0.6 (-0.1-1.2) | 0.5 (0.0-1.0) | 3.2 (-0.3-6.9) | 0.8 (-0.7-2.3) | 0.8 (-7.0-9.2) | 0.6 (-0.1-1.2) |
| lag6 | -0.3 (-1.1-0.5) | -0.1 (-0.7-0.4) | 0.3 (-3.5-4.3) | -1.0 (-2.7-0.6) | -6.6 (-14.9-2.6) | 0.7 (0.0-1.5) |
| lag7 | -1.3 (-2.8-0.1) | -0.9 (-2.0-0.2) | -3.1 (-9.7-4.0) | -3.1 (-6.2-0.1) | -14.4 (-27.6-1.2) | 0.9 (-0.3-2.2) |
| lag01 | -0.3 (-2.6-2.0) | -0.3 (-2.0-1.5) | -7.3 (-18.1-5.0) | 0.6 (-4.7-6.2) | 0.6 (-23.7-32.5) | 0.3 (-2.0-2.7) |
| lag02 | 0.6 (-1.9-3.2) | 0.4 (-1.4-2.4) | -5.4 (-17.8-8.7) | 2.6 (-3.2-8.9) | 7.3 (-21.5-46.7) | 0.5 (-2.1-3.3) |
| lag03 | 1.8 (-0.8-4.6) | 1.4 (-0.5-3.4) | -1.3 (-14.7-14.2) | 5.2 (-1.1-11.8) | 16.3 (-16.5-61.9) | 0.8 (-2.0-3.8) |
| lag04 | 3.0 (0.1-6.0) | 2.4 (0.2-4.5) | 3.4 (-11.5-20.9) | 7.3 (0.5-14.7) | 23.6 (-13.6-76.9) | 1.2 (-1.8-4.4) |
| lag05 | 3.6 (0.5-6.7) | 2.9 (0.6-5.2) | 6.7 (-9.6-26.0) | 8.2 (0.9-16.0) | 24.6 (-15.0-82.7) | 1.8 (-1.5-5.2) |
| lag06 | 3.3 (0.2-6.4) | 2.7 (0.5-5.1) | 7.1 (-9.8-27.0) | 7.1 (-0.1-14.7) | 16.4 (-21.5-72.6) | 2.6 (-0.9-6.1) |
| lag07 | 1.9 (-1.4-5.3) | 1.8 (-0.7-4.3) | 3.8 (-13.8-24.9) | 3.7 (-3.3-11.3) | -0.3 (-35.0-52.8) | 3.5 (-0.3-7.5) |

Abbreviations: PM_2.5_, particles with aerodynamic diameter <2.5 µm; PM_10_, particles with aerodynamic diameter <10 µm; SO_2_, sulfur dioxide; NO_2_, nitrogen dioxide; CO, carbon monoxide; O_3_, ozone.

Table S2. Percentage changes (excess risk, ER (%)) with 95%CI in ischemic stroke hospitalizations associated with each pollutant’s concentration, by different degree of freedom (df) for calendar time, temperature, and relative humidity.

|  |  | PM_2.5_ | PM_10_ | SO_2_ | NO_2_ | O_3_ |
| --- | --- | --- | --- | --- | --- | --- |
| Time | 6 | 4.0 (0.9-7.2) | 3.2 (0.9-5.5) | 5.5 (-11-25.1) | 7.3 (0.0-15) | 4.3 (-0.6-8.1) |
|  | 7 | 3.6 (0.5-6.7) | 2.9 (0.6-5.2) | 7.1 (-9.8-27) | 8.2 (0.9-16) | 3.5 (-0.3-7.5) |
|  | 8 | 3.5 (0.4-6.7) | 2.8 (0.5-5.2) | 8.1 (-9.2-28.7) | 8.8 (1.4-16.8) | 3.6 (-0.1-7.5) |
| Temperature | 4 | 3.2 (0.1-6.3) | 2.5 (0.2-4.9) | 5.7 (-11-25.4) | 7.4 (0.1-15.3) | 2.4 (-1.7-6.7) |
|  | 5 | 3.2 (0.1-6.4) | 2.5 (0.2-4.9) | 5.4 (-11.2-25) | 8.0 (0.5-16) | 2.5 (-1.6-6.8) |
|  | 6 | 2.7 (-0.4-5.9) | 2.2 (-0.1-4.6) | 4.4 (-12-23.8) | 6.6 (-0.8-14.6) | 2.1 (-2.1-6.4) |
| Relative humidity | 4 | 3.2 (0.1-6.3) | 2.6 (0.3-4.9) | 6.4 (-10.3-26.3) | 7.7 (0.5-15.5) | 3.4 (-0.5-7.3) |
|  | 5 | 3.2 (0.1-6.3) | 2.6 (0.3-4.9) | 6.4 (-10.3-26.2) | 7.7 (0.4-15.5) | 3.3 (-0.5-7.3) |
|  | 6 | 3.2 (0.1-6.4) | 2.6 (0.3-4.9) | 6.3 (-10.4-26.2) | 7.7 (0.4-15.5) | 3.3 (-0.5-7.3) |

Abbreviations: PM_2.5_, particles with aerodynamic diameter <2.5 µm; PM_10_, particles with aerodynamic

diameter <10 µm; SO_2_, sulfur dioxide; NO_2_, nitrogen dioxide; O_3_, ozone.
